# The Putative Mevalonate Diphosphate Decarboxylase from Picrophilus torridus Is in Reality a Mevalonate-3-Kinase with High Potential for Bioproduction of Isobutene

**DOI:** 10.1128/AEM.04033-14

**Published:** 2015-03-12

**Authors:** Luca Rossoni, Stephen J. Hall, Graham Eastham, Peter Licence, Gill Stephens

**Affiliations:** a Department of Chemical and Environmental Engineering, Biorenewables and Bioprocessing Research Group, University of Nottingham, Nottingham, United Kingdom; b Lucite International, Wilton Centre, Wilton, United Kingdom; c School of Chemistry, University of Nottingham, Nottingham, United Kingdom

## Abstract

Mevalonate diphosphate decarboxylase (MVD) is an ATP-dependent enzyme that catalyzes the phosphorylation/decarboxylation of (*R*)-mevalonate-5-diphosphate to isopentenyl pyrophosphate in the mevalonate (MVA) pathway. MVD is a key enzyme in engineered metabolic pathways for bioproduction of isobutene, since it catalyzes the conversion of 3-hydroxyisovalerate (3-HIV) to isobutene, an important platform chemical. The putative homologue from Picrophilus torridus has been identified as a highly efficient variant in a number of patents, but its detailed characterization has not been reported. In this study, we have successfully purified and characterized the putative MVD from P. torridus. We discovered that it is not a decarboxylase *per se* but an ATP-dependent enzyme, mevalonate-3-kinase (M3K), which catalyzes the phosphorylation of MVA to mevalonate-3-phosphate. The enzyme's potential in isobutene formation is due to the conversion of 3-HIV to an unstable 3-phosphate intermediate that undergoes consequent spontaneous decarboxylation to form isobutene. Isobutene production rates were as high as 507 pmol min^−1^ g cells^−1^ using Escherichia coli cells expressing the enzyme and 2,880 pmol min^−1^ mg protein^−1^ with the purified histidine-tagged enzyme, significantly higher than reported previously. M3K is a key enzyme of the novel MVA pathway discovered very recently in Thermoplasma acidophilum. We suggest that P. torridus metabolizes MVA by the same pathway.

## INTRODUCTION

The mevalonate (MVA) pathway (1) is the main route for the production of isopentenyl pyrophosphate (IPP) from acetyl coenzyme A (acetyl-CoA) in a diverse cohort of organisms, including higher eukaryotes, archaea, a few eubacteria, fungi, and plants (2). IPP is a key building block for a large family of metabolites such as isoprenoids, dolichols, and sterols that are crucial for a wide variety of cellular functions (3). Many of these molecules have found applications in medicine and agriculture or are used as nutraceuticals, flavors, and fragrances (4, 5). Inhibition of the MVA pathway may also be a target for the design of antibacterial drugs (6).

More recently, the emergence of industrial synthetic biology has led to more and more examples of metabolic engineering using enzymes from the MVA pathway for sustainable production of bioactive and industrial chemicals (7). Products include amorpha-4,11-diene (the precursor to artemisinin) (8, 9), limonene and β-carotene (10–12), a range of sesquiterpenes with potential applications in cosmetics, pharmaceuticals, and jet fuels (13–15), and other biofuels, such as prenol, isoprenol, or other C_5_ compounds (16, 17).

**FIG 1:**
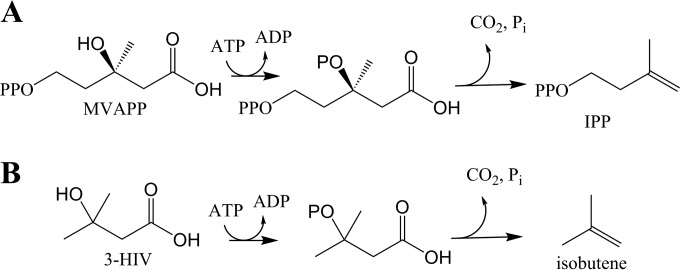
MVD reaction scheme. (A) The natural substrate of MVD is (*R*)-mevalonate-5-diphosphate (MVAPP), which is converted to isopentenyl pyrophosphate (IPP). (B) MVD is also active on 3-hydroxyisovalerate (3-HIV) to produce isobutene in S. cerevisiae (18).

A noteworthy example is the use of mevalonate diphosphate decarboxylase (EC 4.1.1.33) (MVD) to produce isobutene. In the classical MVA pathway, MVD catalyzes the final step, where it produces IPP from (*R*)-mevalonate-5-diphosphate (MVAPP) in an irreversible reaction dependent upon ATP (Fig. 1A). MVAPP is phosphorylated first, and consequent decarboxylation occurs with the concomitant release of inorganic phosphate. With the same mechanism, classical MVDs also catalyze the conversion of the nonphosphorylated 3-hydroxyisovalerate (3-HIV) to isobutene (Fig. 1B) (18). Isobutene is a small, highly reactive molecule, used extensively as a platform chemical to manufacture a wide variety of products (19, 20) including fuel additives, rubbers, and speciality chemicals. The global demand for isobutene produced from petrochemical sources was estimated to be around 10 million metric tons per year with a market value higher than 15 billion euros (21, 22). Sustainable conversion of renewable feedstocks to isobutene using engineered pathways containing MVD is, therefore, an important target, although current microbial processes are still unsatisfactory (20) compared with current petrochemical production. Nevertheless, Global Bioenergies (Evry, France) has recently announced a second pilot plant study (23).

The first demonstration of isobutene production via MVD employed Escherichia coli engineered with the Saccharomyces cerevisiae mevalonate diphosphate decarboxylase homologue (*Sc*MVD) to catalyze the conversion of 3-HIV (18). A patent regarding the production of 3-HIV from acetone and acetyl-CoA was filed subsequently (24), followed by further patents on the utilization of MVDs from different sources to produce isobutene more efficiently (25, 26). The concept was also extended to production of other molecules such as isoprenol (27, 28).

Among the enzymes tested in the patents, one of the most efficient decarboxylases for isobutene production (25, 27) is the enzyme from Picrophilus torridus (GenBank [29] accession no. AAT43941; locus tag PTO1356), which has been annotated as an MVD (29, 30). The utilization of the putative P. torridus MVD (^put^*Pt*MVD) alone resulted in greater production titers, but even higher yields were obtained when ^put^*Pt*MVD was mixed with MVDs from other organisms (26, 28). To explain this, it has been proposed that ^put^*Pt*MVD catalyzes phosphorylation of the substrate faster than the subsequent decarboxylation. It has further been proposed that the phosphorylated intermediate is released and the additional mixture of other MVDs catalyzes decarboxylation of this intermediate to isobutene, providing an overall increase in efficiency (26, 28). However, this hypothesis remains to be tested, since ^put^*Pt*MVD has not yet been purified and characterized, and the reaction mechanism has not been confirmed.

In the interest of unveiling more information on this poorly characterized enzyme and its potential for isobutene formation, we investigated ^put^*Pt*MVD in more detail. After sequence analysis, ^put^*Pt*MVD was tested on the putative natural substrate MVAPP and on other potential substrates. We found that the enzyme is not an MVD. Instead, it belongs to a new class of enzyme, mevalonate-3-kinase (M3K), very recently discovered in Thermoplasma acidophilum (31, 32). Finally, ^put^*Pt*MVD's ability to produce isobutene from 3-HIV and its potential to produce other valuable small alkenes were investigated. Isobutene production rates were significantly higher than reported previously (18).

This study provides important insights into a new enzyme involved in the production of isoprenoids. Since M3Ks belong to a new class of enzymes, there is considerable scope to discover new biocatalysts with potential industrial uses. In particular, M3K catalyzes isobutene formation at a higher rate than other biocatalysts.

## MATERIALS AND METHODS

### Materials.

Bacterial strains and plasmids were purchased from Novagen. Enzymes for gene cloning and strain transformation were purchased from Fermentas, while purification kits for genes and plasmids were purchased from Qiagen. Materials for protein purification and SDS-PAGE were purchased from GE Healthcare and Bio-Rad. 3-Hydroxyisovalerate (3-HIV) was purchased from TCI Chemicals, and 3-hydroxypropionate was purchased from Manchester Organics. All other chemicals were purchased from Sigma-Aldrich unless stated otherwise.

### Sequence and structure analysis.

Sequences were searched with BLASTp (33) using the ^put^*Pt*MVD amino acid sequence (GenBank [29] accession no. AAT43941) within nonredundant sequences database (November 2014). The E value cutoff was set at 0.1, and the 47 most significant hits were aligned with MUSCLE (34). Phylogenetic reconstruction was performed with MEGA6 (35) by the maximum likelihood test.

For multiple-sequence alignment of classical MVDs and Thermoplasmatales putative MVDs, amino acid sequences used were from the following organisms (with the respective GenBank [29] accession no. shown in parentheses): Staphylococcus epidermidis (AAO03959), Saccharomyces cerevisiae (AAC49252), Legionella pneumophila (AAU28109), Trypanosoma brucei (EAN78728), Candida albicans (AAF19399), Homo sapiens (AAC50440), Mus musculus (CAC35731), Rattus norvegicus (AAB00192), Arabidopsis thaliana (AEE79204), Ginkgo biloba (AAV32433), Staphylococcus aureus (BAB56753), Streptococcus pyogenes (AAK33797), Streptococcus pneumoniae (CCP34068), Thermoplasma volcanium (BAB59465), Thermoplasma acidophilum (CAC12426), Ferroplasma acidarmanus (AGO61795), and Picrophilus torridus (AAT43941). Alignment was generated using MUSCLE (34) and rendered with ESPript (36).

### Cloning.

Genes encoding ^put^*Pt*MVD and *Sc*MVD, optimized for expression in E. coli (see Fig. S2 and Fig. S3 in the supplemental material), were synthesized by Biomatik with the inclusion of NdeI and NotI restriction sites to facilitate insertion into a modified version of plasmid pET-20b(+), adapted for N-terminal histidine tagging. Escherichia coli BL21(DE3)pLysS was transformed by heat shock, and the transformants were selected on LB agar plates supplemented with carbenicillin (50 µg/ml), chloramphenicol (34 µg/ml), and glucose (10 g/liter). E. coli was also transformed with the empty plasmid and used as a control. Glycerol stocks (8%) of transformants were stored at −80°C, and experimental cultures were derived from single colonies grown overnight on LB agar at 37°C. For expression or whole-cell biotransformation assays, single colonies from those plates were used to inoculate precultures.

### Expression and purification.

Overnight precultures (50 ml) of E. coli strains were grown in LB medium (500 ml) with antibiotics and incubated at 37°C with shaking at 160 rpm. At an optical density at 600 nm (OD_660_) of approximately 0.6, protein expression was induced by adding isopropyl-β-d-1-thiogalactopyranoside (IPTG) (0.4 mM). After 14 h, cells were removed by centrifugation and resuspended in buffer A (20 mM sodium phosphate, 30 mM imidazole, 500 mM NaCl [pH 7.4]) for subsequent purification or in buffer B (50 mM Tris-HCl, 10 mM MgCl_2_, 20 mM KCl [pH 7.5]) when the supernatants were to be used directly as crude extracts. The cells were lysed with a Constant Systems cell disruption system, cell debris was removed by centrifugation at 15,000 × *g* for 15 min, and protein expression was checked by SDS-PAGE (see below).

^put^*Pt*MVD and *Sc*MVD were purified at 4°C using an Akta protein purification system. Crude extracts (10 ml) were applied to a HisTrap FF crude column (1 ml), and proteins were eluted with a linear gradient of buffer A containing 500 mM imidazole (1 ml/min). Elution was monitored by UV (280 nm). Fractions (2 ml) were analyzed by SDS-PAGE (see below), and those containing the protein of interest were combined. Buffer A was exchanged with buffer B by several concentration/dilution cycles using a Vivaspin sample concentrator. Fresh enzymes were used each time.

SDS-PAGE was done with a Mini-Protean electrophoresis system using TGX precast gels stained with QC colloidal Coomassie protein stain. Purity was estimated by visual observation. Total protein concentration in crude extracts was determined with the Bio-Rad DC protein assay kit. The concentrations of pure ^put^*Pt*MVD and *Sc*MVD were calculated from *A*_280_ measurements with a NanoDrop ND1000 spectrophotometer (Thermo Scientific) using a computed extinction coefficient of 22,015 and 53,650 M^−1^ · cm^−1^, respectively.

### Enzyme assays.

The activity of purified ^put^*Pt*MVD and *Sc*MVD was determined by coupling the release of ADP with NADH oxidation by pyruvate kinase/lactate dehydrogenase (PK/LDH) (37–41), measured at 340 nm at 10-s intervals using an Agilent 8453 UV-visible spectrophotometer. Assays were performed in 1-ml polymethyl methacrylate cuvettes (BRAND) at 30°C in buffer B with the addition of 0.2 mM NADH, 0.5 mM phosphoenolpyruvate (PEP), 5 mM ATP, aqueous glycerol solution of PK/LDH (20 μl) (catalog no. P0294; Sigma-Aldrich), and 10 to 50 μg of pure enzyme. Assays were equilibrated for 2 min and initiated by the addition of substrate (see below). NADH concentrations were calculated using an extinction coefficient of 6,220 M^−1^ · cm^−1^. One unit of enzyme activity corresponds to production of 1 μmol NADH per 1 min.

### Substrate range and kinetic characterization.

^put^*Pt*MVD and *Sc*MVD were tested for activity with (*R*,*S*)-mevalonate-5-diphosphate (MVAPP), (*R*,*S*)-mevalonate-5-phosphate (MVA-5P), and (*R*)-mevalonate (MVA), all at 500 μM. ^put^*Pt*MVD was also tested for activity with 3-hydroxyisovalerate (3-HIV), 3-hydroxybutyrate (3-HB), and 3-hydroxypropionate (3-HP), at the same concentration.

For ^put^*Pt*MVD kinetic characterization, the MVA concentration range was 75 to 900 μM, and the 3-HIV and 3-HB concentrations range from 1 to 25 mM. For *Sc*MVD kinetic characterization, the MVAPP concentration range was 50 to 300 μM. When performing kinetic analyses for the cosubstrate ATP, MVA and MVAPP were kept in excess (2 mM) and ATP was kept in a range between 18.75 and 300 μM. Maximum velocities (*V*_max_) and Michaelis constants (*K_m_*) were estimated using the Michaelis-Menten model of GraphPad Prism version 6.04.

### Product identification.

For isoprenol analysis by gas chromatography-mass spectrometry (GC-MS) and phosphorylated MVA identification by electrospray ionization-mass spectrometry (ESI-MS), products were generated in reaction mixtures (1 ml) containing 5 mM MVA, 6 mM ATP, and 50 μg pure ^put^*Pt*MVD in buffer B at 30°C with shaking at 200 rpm. Incubation times are stated in the text. Control samples were prepared in the same way but without adding the enzyme.

Enzymatic reaction mixtures for nuclear magnetic resonance (NMR) were incubated for 24 h at 30°C with shaking at 200 rpm in reaction mixtures described previously (31) but using a larger volume (300 μl) and a higher concentration of MVA and PEP (40 mM). Analytical standards of MVA and MVA-5P were prepared at a concentration of 40 mM.

### Formation of isobutene and other gaseous alkenes.

Isobutene formation was investigated for both ^put^*Pt*MVD and *Sc*MVD using whole cells, crude extracts, and pure enzymes. Isobutene formation was linear until 48 h, so production rates were calculated during this period. ^put^*Pt*MVD was also tested for ethene and propene formation from 3-HP and 3-HB, respectively, in crude extracts and with purified enzyme. Quantification of all gases was performed by GC-MS.

### (i) Whole-cell assays.

E. coli strains were grown and induced as described above. After 5 h, cells were collected by centrifugation at 15,000 × *g* for 15 min and resuspended in fresh medium containing IPTG. Reaction mixtures (10 ml) containing harvested cells (20 g/liter) and 3-HIV (50 mM) were incubated at 37°C with shaking at 200 rpm in 30-ml glass vials closed with gas-tight caps with polytetrafluorothylene (PTFE)-silicone septa.

### (ii) Crude extract and pure enzyme assays.

Reaction mixtures (1 ml) containing crude extracts (1 mg/ml total protein) or pure ^put^*Pt*MVD and *Sc*MVD (50 μg), ATP (40 mM), and either 3-HIV, 3-HB, or 3-HP (40 mM) in buffer B were incubated at 30°C with shaking at 200 rpm in sealed GC vials (nominal volume of 2 ml) with PTFE screw caps. For controls, samples without the addition of enzymes were also analyzed. The reaction mixtures were incubated at the temperatures stated in the text.

### Analytical methods. (i) GC-MS.

Samples were analyzed using an Agilent 7890A GC-MS system equipped with a Restek Rxi-5ms capillary column (0.25 mm by 30 m by 0.25 μm) and a 5975C inert mass selective detector (MSD) with a quadrupole mass analyzer. Helium was used as a carrier gas at 1.2 ml/min. Data analysis was done using the Agilent MSD ChemStation software and by comparison with the mass spectra and retention times of authentic standards.

For detection and quantification of isoprenol, samples of reaction mixtures (250 μl) were extracted with 750 μl ethyl acetate, and the solvent phase (1 μl) was injected at an inlet temperature of 250°C and at a split ratio of 100:1. The oven temperature was kept at 45°C for 5 min, increased to 300°C at 20°C/min, and then held for 5 min. The retention time of authentic isoprenol was 3.32 min, and a linear calibration curve was obtained over a concentration range from 0.1 to 10 mM.

For detection and quantification of isobutene, propene, and ethene, the headspace (100 μl) was manually injected at an inlet temperature of 150°C and at a split ratio of 100:1. The oven temperature was kept at 40°C for 5 min, increased to 200°C at 20°C/min, and then held for 5 min. The retention times of authentic isobutene, propene, and isobutene were 1.42, 1.38, and 1.34 min, respectively. A linear calibration curve was obtained for all gases over a concentration range from 4,000 to 200,000 pmol/ml. At low product concentrations, sensitivity was increased by monitoring the most characteristic ions in selected ion monitoring (SIM) mode. For isobutene, the ions were 39, 41, and 56 *m/z*; for propene, they were 39, 41, and 42 *m/z*; for ethene, they were 26 and 27 *m/z*.

### (ii) ESI-MS.

Reaction mixtures were filtered using a Vivaspin sample concentrator to remove enzyme and quench the reaction. Filtrates were analyzed by ESI-MS operating in the negative ion mode using a Varian 310-MS triple quadrupole (TQ) system controlled by a Varian Workstation version 6.9. Samples were diluted in 50% water–50% acetonitrile for direct infusion at 0.05 ml/min. Multiple spectra (10 spectra) were averaged to generate the data used in the figures. The masses of the negatively charged ions discussed are mevalonate-3-phosphate (227 *m/z*) and mevalonate (147 *m/z*), and the phosphate species are H_2_PO_4_ (97 *m/z*) and PO_3_ (79 *m/z*). For tandem MS (MS-MS) acquisition, the isolation width was set at 1 *m/z*, and the collision energy was set at 10 eV.

### (iii) NMR.

The samples were diluted to 600 μl with 99.9% D_2_O. Spectra were acquired at ambient temperature using a 500-MHz Bruker AV(III)500 spectrometer equipped with a cryoprobe. Data were processed and analyzed using ACD/NMR Processor version 12.01.

## RESULTS

### Sequence analysis.

**FIG 2:**
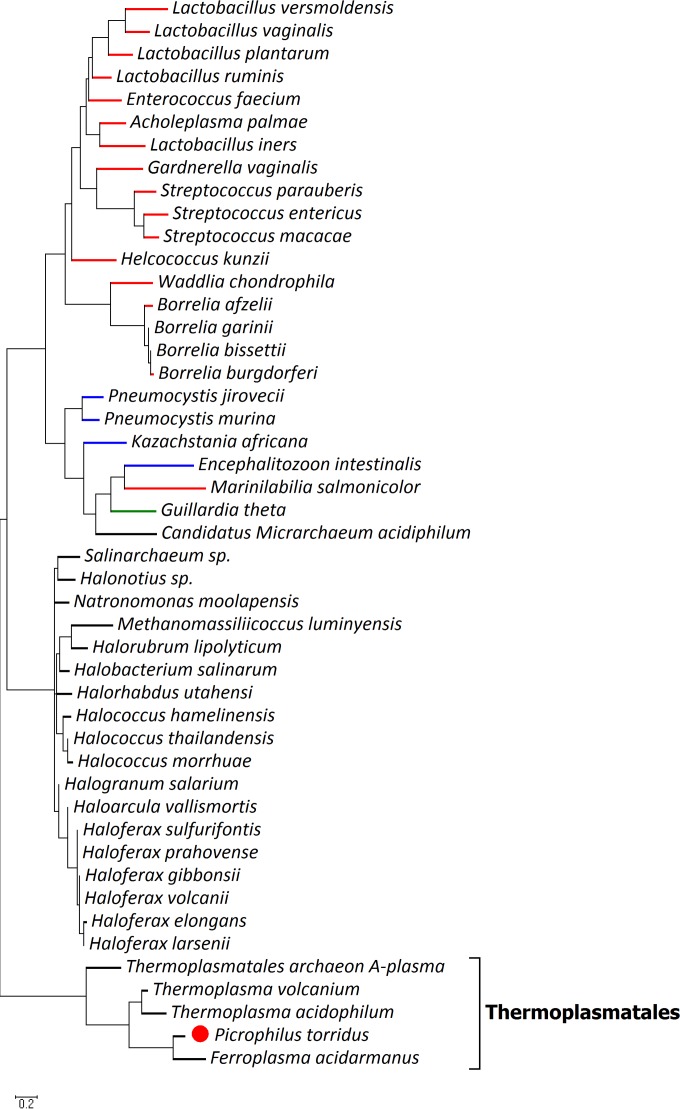
Phylogenetic analysis. P. torridus putative MVD is indicated by a red circle. The color of the line before the organism name in the dendrogram shows the kingdom of the organism as follows: red line, the Bacteria kingdom; blue line, the Fungi kingdom; green line, the Chromalveolata kingdom; black line, the Archaea kingdom. The black bracket indicates archaea belonging to the Thermoplasmatales order.

Initially, a bioinformatics approach was used to gain insights into the possible functionality of ^put^*Pt*MVD. A BLASTp search for ^put^*Pt*MVD homologues and a phylogenetic analysis revealed that proteins with the highest sequence identity and similarity with ^put^*Pt*MVD are found mostly in members of the Archaea domain, with higher values with enzymes from Ferroplasma acidarmanus, Thermoplasma volcanium, and T. acidophilum, all members of the Thermoplasmatales order (Fig. 2; see Table S1 in the supplemental material). Interestingly, ^put^*Pt*MVD has sequence identity and similarity of only 21% and 41%, respectively, with Staphylococcus epidermidis MVD (*Se*MVD) (38), and only 18% and 36% with *Sc*MVD (42), two enzymes that have been verified to act catalytically as MVDs. This significant difference opens the possibility that ^put^*Pt*MVD, and the other Thermoplasmatales homologues, may be unrelated to classical MVDs.

**FIG 3:**
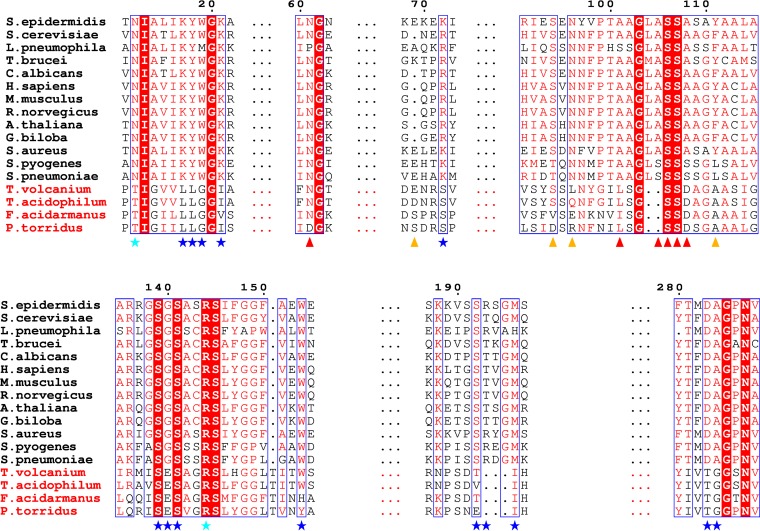
Multiple-sequence alignment. Conventional MVDs are indicated in black, and “MVD-like” proteins from the Thermoplasmatales order are shown in red. Only highly conserved regions (outlined in blue) are depicted. Conservation of residues is denoted by colors: white amino acids on a red background are strictly conserved; red amino acids on a white background are high in similarity. The numbering annotation is based on S. epidermidis MVD. Stars denote residues involved in MVAPP interaction (cyan for C-1 carboxylate group and blue for diphosphate moiety and hydroxyl group). Triangles denote residues involved in ATP interaction (red for phosphate groups, orange for purine ring and ribose).

To further investigate this hypothesis, a multiple-sequence alignment was made between Thermoplasmatales homologues and classical MVDs to compare important conserved regions and residues known to be involved in substrate binding and enzyme activity (Fig. 3). The classical MVDs were chosen on the basis of their experimentally verified ability to catalyze decarboxylation of MVAPP and the availability of structural, functional, and mutation data. The regions for ATP binding and interaction with the C-1 carboxylate of MVAPP are very similar among the whole cohort (Fig. 3, positions 12, 101 to 111, and 144). However, strong differences are seen in those regions particularly suggested to be involved in the binding of the diphosphate moiety of MVAPP or directly involved in the catalytic mechanism (Fig. 3, positions 17 to 21, 72, 139 to 141, 192 to 196, and 283). This information suggests that ^put^*Pt*MVD may differ significantly from classical MVDs (18 to 21% sequence identity). In fact, it shares greater sequence identity (38%) with the newly discovered T. acidophilum mevalonate-3-kinase (*Ta*M3K) (31, 32) (Fig. 3), which was also previously annotated as an MVD. Unlike MVDs, the substrate for *Ta*M3K is (*R*)-mevalonate (MVA), and the enzyme acts as a kinase to produce (*R*)-mevalonate-3-phosphate (MVA-3P) and not as a decarboxylase (31, 32). The crystal structure of *Ta*M3K was very recently described, and the importance of some of the amino acid differences mentioned above in comparison to classical MVDs was confirmed (43).

Nevertheless, the sequence identity between *Ta*M3K and ^put^*Pt*MVD is still relatively low, leaving questions about their functional similarity. Since ^put^*Pt*MVD is an important industrial enzyme, we decided to investigate the substrate specificity and reaction selectivity to identify its real biological function.

### Search for ^put^*Pt*MVD's natural substrate.

The sequence of the gene encoding ^put^*Pt*MVD was optimized for expression in E. coli (see Fig. S2 in the supplemental material), transformed, and expressed in E. coli BL21(DE3)/pLysS with an N-terminal histidine tag (6×His). Overnight expression under IPTG induction lead to synthesis of a protein of around 40 kDa that, after purification on a Ni Sepharose column, was at least 95% pure.

Activity was investigated using a coupled enzymatic assay already in use for the analysis of substrate phosphorylation by classical MVDs (37–41). There was no activity when MVAPP was tested as the substrate. (*R*,*S*)-Mevalonate-5-phosphate (MVA-5P) was also tested, since this is the substrate for another enzyme, MVA-5P decarboxylases (phosphomevalonate decarboxylase [PMD]), similar to MVD, and recently discovered in Archaea and Bacteria (44, 45). However, there was no detectable activity on MVA-5P. Finally, MVA was tested as well, since it is the substrate for *Ta*M3K (31, 32). With MVA, activity was detected at a rate of 3.01 U mg^−1^ (Table 1). Overall, the substrate range is consistent with the hypothesis that ^put^*Pt*MVD is actually an M3K, similar to *Ta*M3K, rather than a classical MVD, since it did not act on MVAPP. As a comparison, the classical *Sc*MVD (sequence available in Fig. S3 in the supplemental material) was expressed and purified in the same way as ^put^*Pt*MVD and tested on the same substrates. As expected, *Sc*MVD activity was observed only with MVAPP and not with MVA-5P or MVA (Table 1).

**TABLE 1:** Kinetic constants of *Sc*MVD, ^put^*Pt*MVD, PMD from H. volcanii and R. castenholzii, and M3K from T. acidophilum^*a*^

Enzyme	Reference(s)	Activity (U · mg^−1^)	*K_m_* (μM)^*b*^	*K*_*m*(ATP)_ (μM)	*k*_cat_ (s^−1^)	*k*_cat_/*K_m_* (s^−1^ · μM^−1^)^*b*^
P. torridus ^put^MVD		3 ± 0.1	131 ± 18 (MVA)	23 ± 0.1	1.9	1.5 × 10^−2^ (MVA)
S. cerevisiae MVD	This study	7 ± 0.7	133 ± 28 (MVAPP)	61 ± 6	5.4	4 × 10^−2^ (MVAPP)
S. cerevisiae MVD	39, 40	6.4 ± 0.2	123 ± 22 (MVAPP)	61 ± 6	4.9	4 × 10^−2^ (MVAPP)
H. volcanii PMD	44	5.6 ± 0.1	159 ± 15 (MVA-5P)	289 ± 15	3.5	2.2 × 10^−2^ (MVA-5P)
R. castenholzii PMD	45	NA	152 ± 38 (MVA-5P)	NA	1.7	1.1 × 10^−2^ (MVA-5P)
T. acidophilum M3K	31	NA	97 ± 6 (MVA)	NA	5.0	5.1 × 10^−2^ (MVA)

a Activity values refer to the rate of ADP formation coupled to NADH oxidation. Values for ^put^*Pt*MVD and *Sc*MVD are calculated from enzyme assays at 30°C in 50 mM Tris-HCl buffer, pH 7.5, with the addition of 10 mM MgCl_2_, 20 mM KCl, and 10 μg of pure ^put^*Pt*MVD or *Sc*MVD. Assays were done in triplicate, and the means ± standard errors of the means for the kinetic constants are shown. Previous data for *Sc*MVD, H. volcanii PMD, R. castenholzii PMD, and T. acidophilum M3K are taken from the literature. NA, not available.

b Values are given for the respective substrate (in parentheses) of each enzyme (MVAPP, MVA-5P, or MVA).

In order to understand whether MVA is ^put^*Pt*MVD's natural substrate, we measured and compared the kinetic parameters for MVA phosphorylation by ^put^*Pt*MVD with MVAPP phosphorylation by *Sc*MVD (Table 1). The kinetic parameters for *Sc*MVD were very similar to those reported previously (Table 1) (39, 40). The magnitude of the kinetic constants and activities of ^put^*Pt*MVD with MVA were similar to those of *Sc*MVD with MVAPP, consistent with the hypothesis that MVA is the natural substrate of ^put^*Pt*MVD, as MVAPP is for *Sc*MVD. In addition, the kinetic parameters reported in the literature for the newly discovered PMDs from Haloferax volcanii (44) and Roseiflexus castenholzii (45), and of M3K from T. acidophilum are also similar in magnitude (Table 1).

### New enzyme classification of ^put^*Pt*MVD.

To discriminate between ^put^*Pt*MVD being an MVD or an M3K, further experiments were needed, since the coupled enzyme assay monitors only ADP release and not the actual decarboxylation of the intermediate. The expected reaction product would be 3-methylbut-3-en-1-ol (isoprenol) if MVA is phosphorylated and then decarboxylated. Therefore, attempts were made to detect isoprenol by gas chromatography-mass spectrometry (GC-MS). Pure ^put^*Pt*MVD was mixed with a high concentration of MVA (5 mM), and the presence of isoprenol was checked at intervals (24, 48, and 96 h). Even after a long incubation time (96 h), isoprenol was not observed, suggesting that ^put^*Pt*MVD does not catalyze MVA decarboxylation *per se*.

**FIG 4:**
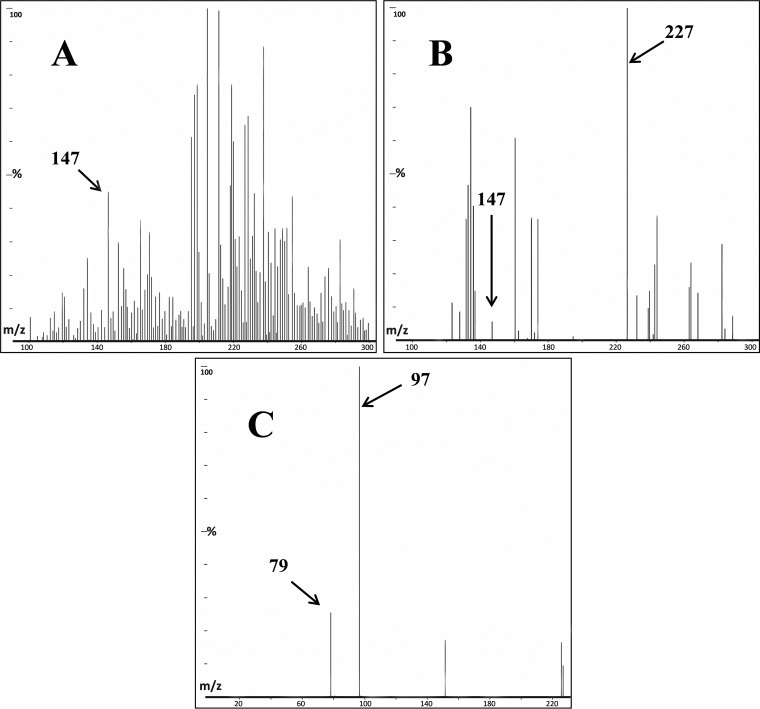
ESI-MS analysis. All spectra were collected in negative ion mode. The mass spectra are shown. (A) Control sample containing MVA but without added enzyme; (B) sample containing MVA and ^put^*Pt*MVD; (C) MS-MS spectrum of 227 *m/z* ion yielding phosphate ions (97 and 79 *m/z*). The reaction was performed in 50 mM Tris-HCl buffer, pH 7.5, with the addition of 10 mM MgCl_2_ and 20 mM KCl. Samples were analyzed after 24 h.

Therefore, we tested ^put^*Pt*MVD for formation of a phosphorylated product from MVA, analyzing the reaction product by electrospray ionization-mass spectrometry (ESI-MS). An ion with *m/z* 227 was detected only in the samples where ^put^*Pt*MVD was added to the reaction mixture, which corresponded to the mass of the expected phosphorylated MVA (Fig. 4B). In the control without added enzyme, this ion was not present. Instead, an ion with 147 *m/z* was among those with the highest intensity, corresponding to unreacted MVA (Fig. 4A). A higher background noise was observed in the MS trace for the control compared to the test. This was probably due to a lower response of MVA in the ESI-MS analysis compared to the phosphorylated MVA. Unreacted MVA was also observed in samples where the enzyme was added, meaning that full conversion was not reached (Fig. 4B). As a confirmation that the 227 *m/z* ion corresponded to phosphorylated MVA, MS-MS analysis of the isolated product generated peaks corresponding to phosphate ions (H_2_PO_4_^−^ [97 *m/z*] and PO_3_^−^ [79 *m*/*z*] [Fig. 4C]).

**FIG 5:**
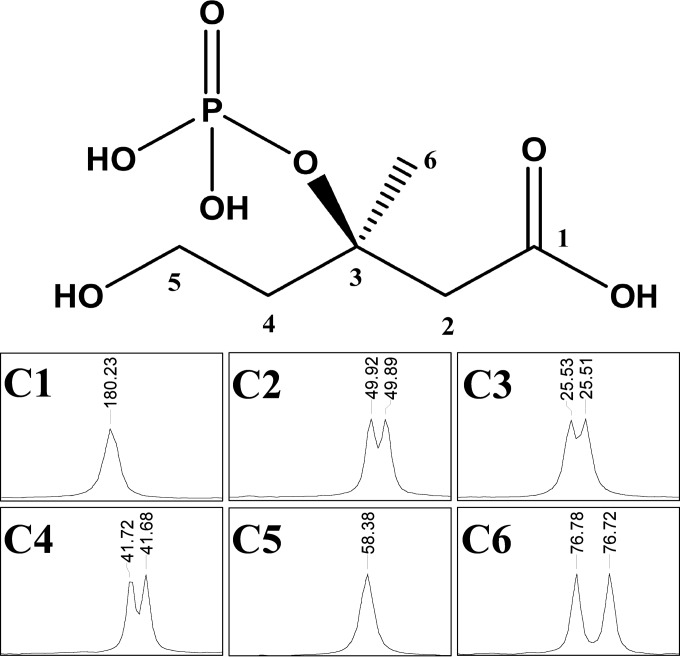
NMR analysis. The full spectrum is available in the supplemental material (see Fig. S6 in the supplemental material). The MVA-3P structure is shown at the top, and the peaks corresponding to carbons 1 to 6 are shown at the bottom. Values are in parts per million (ppm). The reaction was performed as previously described (31) using MVA as the substrate at a concentration of 40 mM. Samples were analyzed after 24 h.

Although the phosphorylated MVA product is likely to be MVA-3P, it must be mentioned that the observed 227 *m/z* ion could also correspond to MVA-5P, the alternative phosphorylation product. Therefore, we used ^13^C NMR analysis to distinguish between the two isomers by analyzing the coupling between ^13^C and ^31^P. Any coupling will result in fine structure (doublets) associated with the signals for each carbon atom within three bonds of ^31^P. The ^13^C NMR spectrum of authentic MVA showed six singlet peaks, whereas the spectrum of authentic MVA-5P showed splitting at carbons 4 and 5 as expected (see Fig. S4 and Fig. S5 in the supplemental material). In contrast, ^13^C NMR spectrum of the product mixture after phosphorylation of MVA by pure ^put^*Pt*MVD showed splitting at carbons 2 to 4 and 6, consistent with phosphorylation at position 3 (Fig. 5; see Fig. S6 in the supplemental material).

**FIG 6:**
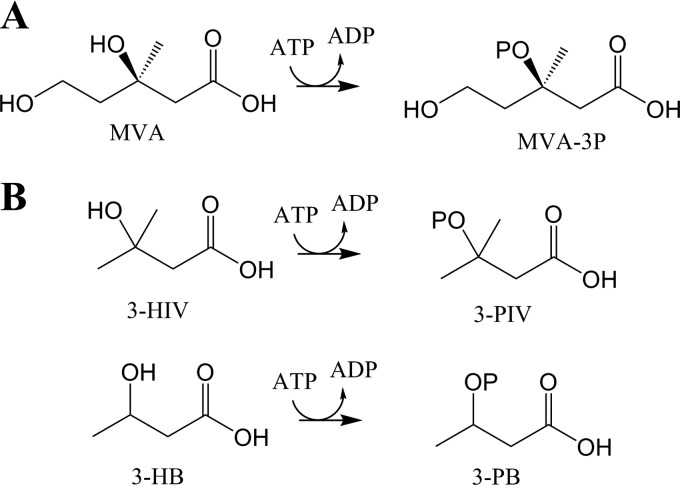
M3K reaction scheme. (A) The natural substrate of M3K is (*R*)-mevalonate (MVA), which is converted to (*R*)-mevalonate-3-phosphate (MVA-3P). (B) *Pt*M3K is also active on 3-hydroxyisovalerate (3-HIV) and 3-hydroxybutyrate (3-HB) to produce 3-phosphoisovalerate (3-PIV) and 3-phosphobutyrate (3-PB), respectively.

Thus, analysis of the sequence, substrate range, and reaction products demonstrated that ^put^*Pt*MVD has been annotated incorrectly and that it is actually a mevalonate-3-kinase that acts on MVA to produce MVA-3P (Fig. 6A). Therefore, we propose that “^put^*Pt*MVD” should be renamed *Pt*M3K. From now on, it is referred to as *Pt*M3K throughout the text.

### Formation of isobutene.

*Pt*M3K was identified as a preferred enzyme for the production of isobutene from 3-HIV (25). Therefore, activity of *Pt*M3K on 3-HIV was first investigated. Compared to the natural substrate MVA, the catalytic efficiency was very low, with a 20-fold-higher *K_m_* and a 1,000-fold-lower *k*_cat_/*K_m_* (Table 2). The *K_m_* and *k*_cat_/*K_m_* were lower (2.35 mM) and higher (4.25 × 10^−5^ s^−1^ μM^−1^), respectively, than those disclosed previously (9.17 mM and 2 × 10^−5^ s^−1^ μM^−1^ [26]).

**TABLE 2:** Kinetic constants of *Pt*M3K on four different substrates^*a*^

Substrate	Activity (U · mg^−1^)	*K_m_* (μM)	*k*_cat_ (s^−1^)	*k*_cat_/*K_m_* (s^−1^ · μM^−1^)
Mevalonate (MVA)	3 ± 0.1	131 ± 18	1.9	1.5 × 10^−2^
3-Hydroxyisovalerate (3-HIV)	0.17 ± 0.01	2,348 ± 574	0.1	4.25 × 10^−5^
3-Hydroxybutyrate (3-HB)	0.14 ± 0.01	3,163 ± 637	0.1	2.9 × 10^−5^
3-Hydroxypropionate (3-HP)	ND	ND	ND	ND

a Activity values refer to the rate of ADP formation coupled to NADH oxidation. Values are calculated at 30°C in 50 mM Tris-HCl buffer, pH 7.5, with the addition of 10 mM MgCl_2_, 20 mM KCl, and 50 μg of pure *Pt*M3K (for MVA, the amount of pure *Pt*M3K added was 10 μg). Assays were done in triplicate, and the means ± standard errors of the means for the kinetic constants are shown. ND, not detected.

Next, isobutene formation was compared using whole cells expressing *Pt*M3K, crude extract containing *Pt*M3K, and pure *Pt*M3K (Table 3). Production rates were compared with *Sc*MVD under the same conditions, since *Sc*MVD also catalyzes isobutene formation from 3-HIV (18). The experiments were performed in sealed vials so that isobutene could be measured by GC-MS analysis of the headspace. Since isobutene can form spontaneously from 3-HIV (46), controls with no enzyme were always compared, and the rate of isobutene formation in controls was subtracted from the enzymatic reactions. As expected (18, 25, 27), both *Pt*M3K and *Sc*MVD catalyzed formation of isobutene (Table 3). In controls without ATP, isobutene formation was similar to those observed for controls without enzyme (data not shown). This confirms that the reactions catalyzed by *Pt*M3K and *Sc*MVD are ATP dependent. *Pt*M3K catalyzed isobutene formation at rates consistently higher than *Sc*MVD under all assay conditions. With the pure enzyme, an 11-fold improvement was observed (Table 3). Very interestingly, a further 110-fold enhancement (from 26 to 2,880 pmol min^−1^ mg protein^−1^) was observed for purified *Pt*M3K when the temperature of the assay was increased from 30 to 50°C (Table 3). However, it must be noted that *Pt*M3K is not a true decarboxylase but a kinase, and therefore, isobutene production depends on formation of an unstable intermediate, 3-phosphoisovalerate (3-PIV) (Fig. 6B) that decarboxylates nonenzymatically. For this reason, it is possible that the higher production rate observed at higher temperature is due to an increased rate of spontaneous decarboxylation of 3-PIV. We wish to note that the rates of isobutene formation obtained using *Pt*M3K were even higher than reported previously using enhanced variants of *Sc*MVD obtained with protein engineering tools (18).

**TABLE 3:** Isobutene formation rates for *Pt*M3K and *Sc*MVD^*a*^

Enzyme	Isobutene formation rate			
	Whole cells (pmol · min^−1^ · g cells^−1^)	Crude extract (pmol · min^−1^ · mg total protein^−1^)	Purified protein (pmol · min^−1^ · mg protein^−1^)	
			30°C	50°C
*Pt*M3K	507 ± 137	261 ± 19	26 ± 2	2,880 ± 140
*Sc*MVD	7.4 ± 1.5	1.6 ± 0.3	2.3 ± 1.2	NA

a The concentration of 3-HIV used was 50 mM. For the crude extract and purified enzyme experiments, 40 mM ATP was added. Assays were done in triplicate, and the means ± standard errors of the means for formation rates are shown. Isobutene was quantified by GC-MS analysis of the headspace. Due to the very low aqueous solubility of isobutene (51), its concentration in the liquid phase was considered negligible, and it was not taken into consideration. NA, not available.

### Formation of other gaseous alkenes.

In order to test the use of *Pt*M3K for production of other small, industrially useful alkenes, we investigated its activity on two other substrates, 3-hydroxypropionate (3-HP) and 3-hydroxybutyrate (3-HB). These substrates have the potential to be converted to ethene and propene (19) if they can be phosphorylated at position 3 and if the intermediates are sufficiently unstable to decarboxylate spontaneously.

Although the enzyme was able to phosphorylate 3-HB (Fig. 6B) with kinetics similar to those for 3-HIV (Table 2), no propene was detected by GC-MS using either crude extract or purified enzyme, even at a temperature of 50°C. This suggests that, as for MVA, the phosphorylated intermediate is too stable to decarboxylate. As for 3-HP, the enzyme was not even able to catalyze its phosphorylation. This suggests that one or two methyl substituents are required at position C-3 to enable binding of the substrate to the enzyme and/or for catalytic activity.

## DISCUSSION

We demonstrated that the P. torridus enzyme (GenBank [29] accession no. AAT43941; locus tag PTO1356), so far classified as mevalonate diphosphate decarboxylase, is actually a mevalonate-3-kinase, since it acted on MVA to produce MVA-3P (Fig. 6A). This intermediate appeared to be stable, since no decarboxylation of MVA was observed and MVA-3P could be detected in the reaction mixture. Classical MVDs also act as kinases, but MVAPP is the substrate instead. The phosphorylation results in formation of a transient tertiary phosphorylated intermediate that quickly releases inorganic phosphate with concomitant decarboxylation (38, 47, 48) or even in a concerted manner (49). Thus, the difference in substrate specificity (MVA versus MVAPP) may account for the differences in the ability of *Pt*M3K and MVDs to act as decarboxylases. Differences in amino acid composition in regions suggested to be involved in the catalytic mechanism (Fig. 3, positions 281 to 284) may also affect the enzymatic mechanism, as recently confirmed for *Ta*M3K (43).

**FIG 7:**
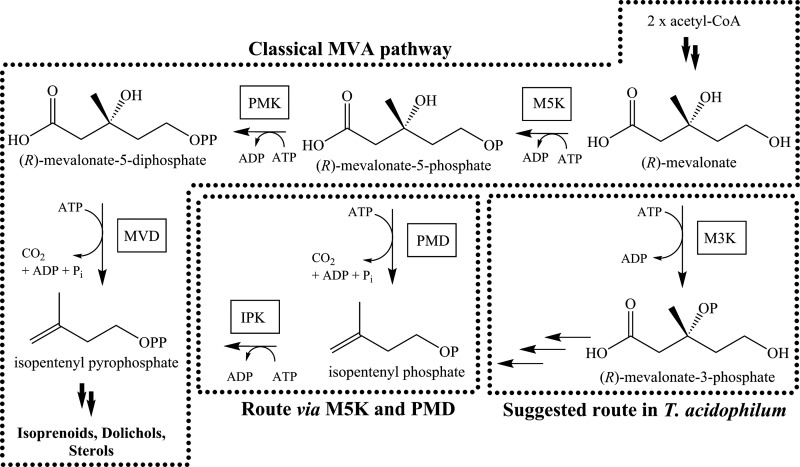
Classical MVA pathway and modified routes. The classical MVA pathway and the modified routes (via M5K and PMD and the newly suggested route in T. acidophilum) are specifically indicated. Enzyme abbreviations are as follows: M5K, mevalonate-5-kinase; PMK, phosphomevalonate (mevalonate-5-phosphate) kinase; MVD, mevalonate-5-diphosphate decarboxylase; PMD, phosphomevalonate (mevalonate-5-phosphate) decarboxylase; IPK, isopentenyl phosphate kinase; M3K, mevalonate-3-kinase.

The results of our sequence analysis suggest that M3Ks may also be found in other members of the Thermoplasmatales order in addition to T. acidophilum (31, 32) and P. torridus. This raises questions about the pathway for MVA metabolism in these organisms, since there is little evidence for the operation of the classical MVA pathway. In the classical MVA pathway, MVA is doubly phosphorylated to MVAPP and consequently converted to IPP by MVD (Fig. 7). In the absence of MVD, MVA must undergo a different route to be converted to IPP. Indeed, the discovery of *Ta*M3K led to the description of a completely new type of MVA pathway in T. acidophilum, where M3K catalyzes the initial step in the metabolism of MVA (31, 32) (Fig. 7) and MVA-3P-5-kinase (M3P5K) (GenBank [29] accession no. CAC11895) catalyzes the conversion of MVA-3P to mevalonate-3,5-bisphosphate (MVA-3,5PP). The enzymes catalyzing conversion of MVA-3,5PP to isopentenyl phosphate (IP) have not yet been identified, and this provides an excellent target for future research. IP is finally converted to IPP via IP kinase (IPK) (50).

We have now shown that an M3K is also present in P. torridus. Since a strongly similar counterpart of M3P5K is also found in P. torridus (see Table S7 in the supplemental material), P. torridus is also likely to contain this new type of MVA pathway (Fig. 7). It should be noted that this pathway is distinct from the mevalonate-5-kinase (M5K) pathway found in Haloferax volcanii (44) and Roseiflexus castenholzii (45) (Fig. 7). Neither of the key enzymes, (M5K and PMD) are present in T. acidophilum (50), and there is no experimental or sequence homology evidence to support their presence in P. torridus. Overall, this suggests that P. torridus produces IPP via the newly discovered M3K pathway.

Regarding the use of *Pt*M3K and MVDs for isobutene production, we found that *Pt*M3K exhibits much higher catalytic efficiency than *Sc*MVD. Unlike *Sc*MVD, the potential of *Pt*M3K in forming the gas is not due to direct decarboxylation but to the generation of the unstable intermediate, 3-PIV (Fig. 6B). The isobutene production rates for *Pt*M3K were not disclosed until now and were found here to be significantly higher than reported previously for *Sc*MVD (18). Having an enzyme with a higher rate of isobutene formation from 3-HIV is an important step for the production of renewable isobutene, since this further reduces the gap toward commercial application. With *Sc*MVD, it was estimated that the level of cellular activity obtained after enhancement of the enzyme with protein engineering (98 pmol min^−1^ g cells^−1^ with whole cells [18]) was about 10^6^-fold below what is needed for a commercial process (18, 20). With *Pt*M3K, the rate is increased 5-fold (507 pmol min^−1^ g cells^−1^). Although this is still far from industrial application, the discovery of a new M3K class of enzymes opens the possibility to screen other, related enzymes for isobutene formation or to undertake protein engineering to improve the production rates still further.

As for the potential of *Pt*M3K to be used for the production of other gaseous alkenes such as ethene and propene, unfortunately, we were not able to observe any product formation. The enzyme was able to phosphorylate 3-HB (Fig. 6B and Table 2), but under the tested conditions, the 3-P intermediate did not spontaneously decarboxylate to propene. This could be due to the 3-phosphobutyrate (3-PB) intermediate being more stable than 3-PIV. The absence of an electron-donating methyl group on 3-PB could reduce the electron density relative to the tertiary carbon of the intermediate and slow down the rate of the decarboxylation, thought to occur via a carbocation intermediate (42).

To conclude, from a microbiological/evolutionary point of view, our study raises important questions on the presence of alternative MVA pathways in Archaea. It is even possible that variant MVA pathways are present among members of all kingdoms in nature (45). Many plant, bacterial, and fungal enzymes are believed to participate in the classical MVA pathway, but like *Pt*M3K, they are still only computationally annotated and need to be investigated experimentally. Discovery of new enzymes is an important goal, since this may yield biocatalysts with improved properties. This can lead to better process efficiencies where production yields with current enzymes are not yet sufficient for commercial viability and where enzyme stability can be a problem. Therefore, discovery of new enzymes of the MVA pathways could aid in improved metabolic engineering for efficient, sustainable bioproduction of chemicals and fuels.

## Supplementary Material

Supplemental material

## References

[1] Bloch K 1965 The biological synthesis of cholesterol. Science 150:19–28. doi:10.1126/science.150.3692.19. 5319508

[2] Kuzuyama T, Seto H 2012 Two distinct pathways for essential metabolic precursors for isoprenoid biosynthesis. Proc Jpn Acad Ser B Phys Biol Sci 88:41–52. doi:10.2183/pjab.88.41. PMC3365244 22450534

[3] Holstein SA, Hohl RJ 2004 Isoprenoids: remarkable diversity of form and function. Lipids 39:293–309. doi:10.1007/s11745-004-1233-3. 15357017

[4] Gershenzon J, Dudareva N 2007 The function of terpene natural products in the natural world. Nat Chem Biol 3:408–414. doi:10.1038/nchembio.2007.5. 17576428

[5] Kirby J, Keasling JD 2009 Biosynthesis of plant isoprenoids: perspectives for microbial engineering. Annu Rev Plant Biol 60:335–355. doi:10.1146/annurev.arplant.043008.091955. 19575586

[6] Wilding EI, Brown JR, Bryant AP, Chalker AF, Holmes DJ, Ingraham KA, Iordanescu S, So CY, Rosenberg M, Gwynn MN 2000 Identification, evolution, and essentiality of the mevalonate pathway for isopentenyl diphosphate biosynthesis in gram-positive cocci. J Bacteriol 182:4319–4327. doi:10.1128/JB.182.15.4319-4327.2000. 10894743 PMC101949

[7] Misawa N 2011 Pathway engineering for functional isoprenoids. Curr Opin Biotechnol 22:627–633. doi:10.1016/j.copbio.2011.01.002. 21310602

[8] Dahl RH, Zhang F, Alonso-Gutierrez J, Baidoo E, Batth TS, Redding-Johanson AM, Petzold CJ, Mukhopadhyay A, Lee TS, Adams PD, Keasling JD 2013 Engineering dynamic pathway regulation using stress-response promoters. Nat Biotechnol 31:1039–1046. doi:10.1038/nbt.2689. 24142050

[9] Yuan J, Ching CB 2014 Combinatorial engineering of mevalonate pathway for improved amorpha-4,11-diene production in budding yeast. Biotechnol Bioeng 111:608–617. doi:10.1002/bit.25123. 24122315

[10] Alonso-Gutierrez J, Chan R, Batth TS, Adams PD, Keasling JD, Petzold CJ, Lee TS 2013 Metabolic engineering of Escherichia coli for limonene and perillyl alcohol production. Metab Eng 19:33–41. doi:10.1016/j.ymben.2013.05.004. 23727191

[11] Kim J-H, Kim S-W, Nguyen DQ-A, Li H, Kim S-B, Seo Y-G, Yang J-K, Chung I-Y, Kim D-H, Kim C-J 2009 Production of β-carotene by recombinant Escherichia coli with engineered whole mevalonate pathway in batch and fed-batch cultures. Biotechnol Bioprocess Eng 14:559–564. doi:10.1007/s12257-008-0230-1.

[12] Jang H-J, Yoon S-H, Ryu H-K, Kim J-H, Wang C-L, Kim J-Y, Oh D-K, Kim S-W 2011 Retinoid production using metabolically engineered Escherichia coli with a two-phase culture system. Microb Cell Fact 10:59. doi:10.1186/1475-2859-10-59. 21801353 PMC3160355

[13] Scalcinati G, Knuf C, Partow S, Chen Y, Maury J, Schalk M, Daviet L, Nielsen J, Siewers V 2012 Dynamic control of gene expression in Saccharomyces cerevisiae engineered for the production of plant sesquiterpene α-santalene in a fed-batch mode. Metab Eng 14:91–103. doi:10.1016/j.ymben.2012.01.007. 22330799

[14] Zhu F, Zhong X, Hu M, Lu L, Deng Z, Liu T 2014 In vitro reconstitution of mevalonate pathway and targeted engineering of farnesene overproduction in Escherichia coli. Biotechnol Bioeng 111:1396–1405. doi:10.1002/bit.25198. 24473754

[15] Wang C, Yoon S-H, Jang H-J, Chung Y-R, Kim J-Y, Choi E-S, Kim S-W 2011 Metabolic engineering of Escherichia coli for α-farnesene production. Metab Eng 13:648–655. doi:10.1016/j.ymben.2011.08.001. 21907299

[16] Gupta D, Summers ML, Basu C 2014 Engineering an isoprenoid pathway in Escherichia coli for production of 2-methyl-3-buten-2-ol: a potential biofuel. Mol Biotechnol 56:516–523. doi:10.1007/s12033-013-9721-1. 24271564

[17] Zheng Y, Liu Q, Li L, Qin W, Yang J, Zhang H, Jiang X, Cheng T, Liu W, Xu X, Xian M 2013 Metabolic engineering of Escherichia coli for high-specificity production of isoprenol and prenol as next generation of biofuels. Biotechnol Biofuels 6:57. doi:10.1186/1754-6834-6-57. 23618128 PMC3654967

[18] Gogerty DS, Bobik TA 2010 Formation of isobutene from 3-hydroxy-3-methylbutyrate by diphosphomevalonate decarboxylase. Appl Environ Microbiol 76:8004–8010. doi:10.1128/AEM.01917-10. 20971863 PMC3008229

[19] Obenaus F, Droste W, Neumeister J 15 6 2000 Butenes. *In* Ullmann's encyclopedia of industrial chemistry. Wiley-VCH Verlag GmbH & Co. KGaA, Weinheim, Germany. doi:10.1002/14356007.a04_483.

[20] van Leeuwen BNM, Wulp AM, Duijnstee I, Maris AJA, Straathof AJJ 2012 Fermentative production of isobutene. Appl Microbiol Biotechnol 93:1377–1387. doi:10.1007/s00253-011-3853-7. 22234536 PMC3275743

[21] de Guzman D 29 7 2011 Rubber industry seeks bio-based chemicals potential. ICIS Chemical Business, London, England http://www.icis.com/Articles/2011/07/01/9481223/Rubber-industry-seeks-bio-based-chemicals-potential.html.

[22] Organization for Economic Cooperation and Development. 2003 Screening Information Data Set (SIDS) initial assessment report: isobutylene. CAS no. 115-11-7. Organization for Economic Cooperation and Development (OECD), Paris, France http://www.inchem.org/documents/sids/sids/115117.pdf.

[23] Global Bioenergies 2014 Global Bioenergies and Fraunhofer CBP take the next step towards the set-up of the Leuna industrial pilot. Global Bioenergies, Evry, France http://www.global-bioenergies.com/communiques/140703_pr_en.pdf.

[24] Marliere P 3 2011 Method for the enzymatic production of 3-hydroxy-3-methylbutyric acid from acetone and acetyl-CoA. Patent WO2011032934A1.

[25] Marliere P January 2010 Production of alkenes by enzymatic decarboxylation of 3-hydroxyalkanoic acids. Patent WO2010001078A2.

[26] Anissimova M, Delcourt M, Marliere P, Tallon R April 2012 Production of alkenes by combined enzymatic conversion of 3-hydroxyalkanoic acids using different mevalonate pyrophosphate decarboxylases. Patent WO2012052427A1.

[27] Marliere P, Anissimova M, Chayot R, Delcourt M June 2011 Process for the production of isoprenol from mevalonate employing a diphosphomevalonate decarboxylase. Patent WO2011076261A1.

[28] Delcourt M, Anissimova M, Marliere P October 2013 Enzymatic production of isoprenol from mevalonate by diphosphomevalonate decarboxylases. Patent WO2013150100A1.

[29] Benson DA, Cavanaugh M, Clark K, Karsch-Mizrachi I, Lipman DJ, Ostell J, Sayers EW 2013 GenBank. Nucleic Acids Res 41:D36–D42. doi:10.1093/nar/gks1195. 23193287 PMC3531190

[30] UniProt Consortium. 2014 Activities at the Universal Protein Resource (UniProt). Nucleic Acids Res 42:D191–D198. doi:10.1093/nar/gkt1140. 24253303 PMC3965022

[31] Vinokur JM, Korman TP, Cao Z, Bowie JU 2014 Evidence of a novel mevalonate pathway in archaea. Biochemistry 53:4161–4168. doi:10.1021/bi500566q. 24914732 PMC4081127

[32] Azami Y, Hattori A, Nishimura H, Kawaide H, Yoshimura T, Hemmi H 2014 (*R*)-Mevalonate 3-phosphate is an intermediate of the mevalonate pathway in Thermoplasma acidophilum. J Biol Chem 289:15957–15967. doi:10.1074/jbc.M114.562686. 24755225 PMC4047369

[33] Altschul SF, Madden TL, Schaffer AA, Zhang J, Zhang Z, Miller W, Lipman DJ 1997 Gapped BLAST and PSI-BLAST: a new generation of protein database search programs. Nucleic Acids Res 25:3389–3402. doi:10.1093/nar/25.17.3389. 9254694 PMC146917

[34] Edgar RC 2004 MUSCLE: multiple sequence alignment with high accuracy and high throughput. Nucleic Acids Res 32:1792–1797. doi:10.1093/nar/gkh340. 15034147 PMC390337

[35] Tamura K, Stecher G, Peterson D, Filipski A, Kumar S 2013 MEGA6: Molecular Evolutionary Genetics Analysis version 6.0. Mol Biol Evol 30:2725–2729. doi:10.1093/molbev/mst197. 24132122 PMC3840312

[36] Gouet P, Robert X, Courcelle E 2003 ESPript/ENDscript: extracting and rendering sequence and 3D information from atomic structures of proteins. Nucleic Acids Res 31:3320–3323. doi:10.1093/nar/gkg556. 12824317 PMC168963

[37] Barta ML, McWhorter WJ, Miziorko HM, Geisbrecht BV 2012 Structural basis for nucleotide binding and reaction catalysis in mevalonate diphosphate decarboxylase. Biochemistry 51:5611–5621. doi:10.1021/bi300591x. 22734632 PMC4227304

[38] Barta ML, Skaff DA, McWhorter WJ, Herdendorf TJ, Miziorko HM, Geisbrecht BV 2011 Crystal structures of Staphylococcus epidermidis mevalonate diphosphate decarboxylase bound to inhibitory analogs reveal new insight into substrate binding and catalysis. J Biol Chem 286:23900–23910. doi:10.1074/jbc.M111.242016. 21561869 PMC3129171

[39] Krepkiy DV, Miziorko HM 2005 Investigation of the functional contributions of invariant serine residues in yeast mevalonate diphosphate decarboxylase. Biochemistry 44:2671–2677. doi:10.1021/bi0484217. 15709780

[40] Krepkiy DV, Miziorko HM 2004 Identification of active site residues in mevalonate diphosphate decarboxylase: implications for a family of phosphotransferases. Protein Sci 13:1875–1881. doi:10.1110/ps.04725204. 15169949 PMC2279928

[41] Alvear M, Jabalquinto AM, Eyzaguirre J, Cardemil E 1982 Purification and characterization of avian liver mevalonate-5-pyrophosphate decarboxylase. Biochemistry 21:4646–4650. doi:10.1021/bi00262a020. 6814481

[42] Dhe-Paganon S, Magrath J, Abeles RH 1994 Mechanism of mevalonate pyrophosphate decarboxylase: evidence for a carbocationic transition state. Biochemistry 33:13355–13362. doi:10.1021/bi00249a023. 7947744

[43] Vinokur JM, Korman TP, Sawaya MR, Collazo M, Cascio D, Bowie JU 2015 Structural analysis of mevalonate-3-kinase provides insight into the mechanisms of isoprenoid pathway decarboxylases. Protein Sci 24:212–220. doi:10.1002/pro.2607. 25422158 PMC4315659

[44] Vannice JC, Skaff DA, Keightley A, Addo JK, Wyckoff GJ, Miziorko HM 2014 Identification in Haloferax volcanii of phosphomevalonate decarboxylase and isopentenyl phosphate kinase as catalysts of the terminal enzyme reactions in an archaeal alternate mevalonate pathway. J Bacteriol 196:1055–1063. doi:10.1128/JB.01230-13. 24375100 PMC3957691

[45] Dellas N, Thomas ST, Manning G, Noel JP 2013 Discovery of a metabolic alternative to the classical mevalonate pathway. eLife 2:e00672. doi:10.7554/eLife.00672. 24327557 PMC3857490

[46] Pressman D, Lucas HJ 1940 The hydration of unsaturated compounds. VIII. The rate of hydration of β,β-dimethylacrylic acid: the rates of dehydration and decarboxylation of β-hydroxyisovaleric acid. J Am Chem Soc 62:2069–2080.

[47] Byres E, Alphey MS, Smith TK, Hunter WN 2007 Crystal structures of Trypanosoma brucei and Staphylococcus aureus mevalonate diphosphate decarboxylase inform on the determinants of specificity and reactivity. J Mol Biol 371:540–553. doi:10.1016/j.jmb.2007.05.094. 17583736

[48] Voynova N, Fu Z, Battaile K, Herdendorf T, Kim J, Miziorko H 2008 Human mevalonate diphosphate decarboxylase: characterization, investigation of the mevalonate diphosphate binding site, and crystal structure. Arch Biochem Biophys 480:58–67. doi:10.1016/j.abb.2008.08.024. 18823933 PMC2709241

[49] Lefurgy ST, Rodriguez SB, Park CS, Cahill S, Silverman RB, Leyh TS 2010 Probing ligand-binding pockets of the mevalonate pathway enzymes from Streptococcus pneumoniae. J Biol Chem 285:20654–20663. doi:10.1074/jbc.M109.098350. 20404339 PMC2898321

[50] Chen M, Poulter CD 2010 Characterization of thermophilic archaeal isopentenyl phosphate kinases. Biochemistry 49:207–217. doi:10.1021/bi9017957. 19928876 PMC3856865

[51] Yaws CL 2003 Yaws' handbook of thermodynamic and physical properties of chemical compounds. Knovel Corporation, New York, NY.

