# Supplementary material for: The Putative Mevalonate Diphosphate Decarboxylase from Picrophilus torridus Is in Reality a Mevalonate-3-Kinase with High Potential for Bioproduction of Isobutene
Source: Appl Environ Microbiol. 2015 Mar 12;81(7):2625–34. doi: 10.1128/AEM.04033-14 (PMC4357925; doi:10.1128/AEM.04033-14)
Supplement: Supplemental material [file supp_81_7_2625__index.html]

Supplemental material 

# The Putative Mevalonate Diphosphate Decarboxylase from Picrophilus torridus Is in Reality a Mevalonate-3-Kinase with High Potential for Bioproduction of Isobutene

## Supplemental material

**Files in this Data Supplement:**

- Supplemental file 1 -

  Search for homologues of put*Pt*MVD(Table S1), optimized gene sequences encoding put*Pt*MVD (Fig. S2) and *Sc*MVD (Fig. S3), full 13C NMR spectra of (*R*)-mevalonate (Fig. S4), (*R*,*S*)-mevalonate-5-phosphate (Fig. S5), and the test reaction containing (*R*)-mevalonate and put*Pt*MVD (*Pt*M3K) (Fig. S6), and search for homologues of the newly discovered mevalonate-3-phosphate 5-kinase from *T. acidophilum* (Table S7).

  PDF, 818K
