# Supplementary material for: The Putative Mevalonate Diphosphate Decarboxylase from Picrophilus torridus Is in Reality a Mevalonate-3-Kinase with High Potential for Bioproduction of Isobutene
Source: Appl Environ Microbiol. 2015 Mar 12;81(7):2625–34. doi: 10.1128/AEM.04033-14 (PMC4357925; doi:10.1128/AEM.04033-14)
Supplement: Supplemental material [file AEM.04033-14_zam999116164so1.pdf]

Supplementary Information

Table S1: search for homologues of ^{put}*Pt*MVD. Sequences were searched with BLASTp (1) using the ^{put}*Pt*MVD amino acid sequence (GenBank (2) entry AAT43941) within nonredundant sequences database (July 2014). The E value cut off was 0.1 and among several resulting hits, the 47 most significant were aligned with MUSCLE (3). *Thermoplasmatales* proteins are in bold

	Organism	NCBI Reference	Identity (%)	Similarity (%)	E value
**1**	***Picrophilus torridus***	**YP_024134**	**100**	**100**	**-**
**2**	***Ferroplasma acidarmanus***	**YP_008142696**	**58.02**	**76.23**	**2.00E-131**
**3**	***Thermoplasma volcanium***	**NP_110839**	**42.86**	**66.46**	**1.00E-83**
**4**	***Thermoplasma acidophilum***	**NP_394760**	**38.73**	**63.81**	**8.00E-70**
**5**	***Thermoplasmatales archaeon A-plasma***	**WP_021795407**	**53.7**	**75.93**	**3.00E-11**
6	*Lactobacillus versmoldensis*	WP_010623536	22.47	43.99	8.00E-06
7	*Lactobacillus iners*	WP_006736906	22.29	42.52	3.00E-05
8	*Streptococcus entericus*	WP_018367392	22.92	40.86	4.00E-04
9	*Encephalitozoon intestinalis*	XP_003073009	24.76	40.84	5.00E-04
10	*Candidatus Micrarchaeum acidiphilum*	EET89942	23.2	43.89	9.00E-04
11	*Halonotius sp.*	WP_021040093	23.24	41.9	0.001
12	*Salinarchaeum sp.*	YP_008055205	19.57	43.48	0.002
13	*Enterococcus faecium*	WP_002351456	19.03	41.69	0.002
14	*Streptococcus parauberis*	WP_003104536	25.08	42.67	0.002
15	*Marinilabilia salmonicolor*	WP_010661914	24.63	44.83	0.002
16	*Borrelia afzelii*	YP_006931874	23.55	43.43	0.002

17	*Helcococcus kunzii*	WP_005398566	22.57	45.14	0.002
18	*Borrelia garinii*	YP_073129	25	45.67	0.003
19	*Waddlia chondrophila*	YP_003709481	25.47	47.2	0.003
20	*Haloferax elongans*	WP_008324111	22.26	40.85	0.003
21	*Natronomonas moolapensis*	YP_007486922	21.54	40	0.004
22	*Haloferax gibbonsii*	WP_004977100	21.54	41.85	0.006
23	*Gardnerella vaginalis*	YP_003373567	24.86	46.49	0.006
24	*Lactobacillus plantarum*	YP_007986982	20.8	40.67	0.008
25	*Halorubrum lipolyticum*	WP_008006049	24.38	39.67	0.009
26	*Halorhabdus utahensi*	YP_003129610	21.88	39.38	0.01
27	*Borrelia bissettii*	YP_004777918	30.19	47.17	0.012
28	*Methanomassiliicoccus luminyensis*	WP_019177897	21.89	42.42	0.016
29	*Halococcus thailandensis*	WP_007742266	22.39	40.8	0.016
30	*Pneumocystis jirovecii*	CCJ31570	22.71	43	0.017
31	*Haloferax prahovense*	WP_008093241	21.81	41.43	0.018
32	*Halogranum salarium*	WP_009374450	22.56	40.85	0.021
33	*Acholeplasma palmae*	CCV64523	21.45	44.16	0.022
34	*Guillardia theta*	XP_005819249	21.9	39.54	0.023
35	*Halococcus morrhuae*	WP_004055468	21.98	41.49	0.024
36	*Haloferax larsenii*	WP_007540006	21.6	41.05	0.028
37	*Borrelia burgdorferi*	YP_005806868	26.15	47.25	0.031
38	*Halococcus hamelinensis*	WP_007692401	21.47	40.49	0.032
39	*Haloarcula vallismortis*	WP_004518165	22.26	40.24	0.035
40	*Haloferax volcanii*	YP_003535463	21.28	41.03	0.035
41	*Lactobacillus ruminis*	YP_004832120	22.9	44.84	0.036

42	*Lactobacillus vaginalis*	WP_003716957	25.41	42.7	0.05
43	*Streptococcus macacae*	WP_003081606	22.04	41.45	0.055
44	*Kazachstania africana*	XP_003956599	23.45	42.41	0.059
45	*Pneumocystis murina*	EMR10307	21.81	42.68	0.072
46	*Haloferax sulfurifontis*	WP_007273787	21.54	41.85	0.086
47	*Halobacterium salinarum*	YP_001688719	21.9	39.68	0.098

Figure S2: gene sequence encoding putPtMVD. The gene sequence used was an optimized version for expression in *Escherichia coli*, already demonstrated to give higher expression yields (4).

```
1     ATGGAGAACTATAATGTTAA AACCCGTGCATTTCCGACCA TTGGTATTATTCTGCTGGGT GGCATTAGCGACAAAAAAAA
81    CCGTATTCCGCTGCATACCA CCGCAGGTATTGCATATACC GGCATCAATAACGATGTGTA CACCGAAACCAAACTGTATG
161   TGAGCAAAGACGAAAAATGC TATATCGATGGCAAAGAAAT CGATCTGAATAGCGATCGTA GCCCGAGCAAAGTGATCGAT
241   AAATTCAAACATGAAATCCT GATGCGTGTGAATCTGGATG ATGAAAACAACCTGAGCATT GATAGCCGCAATTTTAACAT
321   TCTGAGCGGTAGCAGCGATA GCGGTGCAGCAGCACTGGGT GAATGCATTGAAAGCATCTT CGAGTACAACATCAACATCT
401   TCACCTTTGAAAATGATCTG CAGCGTATTAGCGAAAGCGT TGGTCGTAGCCTGTATGGTG GTCTGACCGTTAATTATGCA
481   AATGGTCGTGAAAGCCTGAC CGAACCGCTGCTGGAACCGG AAGCATTTAACAACTTTACC ATCATCGGTGCCCATTTTAA
561   CATTGATCGCAAACCGAGCA ACGAAATCCACGAAAACATC ATCAAACATGAGAACTATCG CGAACGTATTAAAAGCGCAG
641   AGCGCAAAGCAAAAAAACTG GAAGAACTGAGCCGTAATGC CAACATTAAAGGCATTTTTG AACTGGCAGAAAGCGATACC
721   GTGGAATATCATAAAATGCT GCATGATGTGGGCGTTGATA TTATCAATGACCGCATGGAA AATCTGATTGAACGCGTGAA
801   AGAGATGAAAAACAACTTCT GGAACAGCTATATTGTTACC GGTGGTCCGAATGTTTTTGT GATCACCGAGAAAAAAGATG
881   TGGATAAAGCCATGGAAGGT CTGAATGATCTGTGTGATGA TATTCGTCTGCTGAAAGTTG CAGGTAAACCGCAGGTTATC
961   AGCAAAAACTTCTAATGA
```

Figure S3: gene sequence encoding *Sc*MVD. The gene sequence used was optimized for expression in *E. coli* by the synthesizing company, Biomatik.

```
1    ATGACCGTTTACACCGCGTC TGTGACTGCTCCGGTGAACA TCGCAACTCTGAAATACTGG GGTAAGCGTGATACTAAACT
81   GAACCTGCCGACCAACAGCT CTATCAGCGTTACTCTGAGC CAGGACGATCTGCGTACCCT GACCTCTGCAGCTACTGCGC
161  CGGAATTCGAGCGTGACACC CTGTGGCTGAACGGTGAGCC GCACTCTATCGACAACGAAC GTACCCAGAACTGCCTGCGT
241  GACCTGCGCCAGCTGCGTAA AGAGATGGAATCCAAGGATG CGAGCCTGCCGACTCTGTCT CAGTGGAAACTGCACATCGT
321  TTCTGAGAACAACTTCCCGA CCGCAGCGGGCCTGGCGTCT TCTGCTGCGGGTTTCGCGGC ACTGGTTAGCGCGATTGCGA
401  AACTGTACCAGCTGCCGCAG TCTACCTCTGAGATTTCTCG TATCGCGCGCAAGGGCTCTG GCTCTGCGTGCCGTTCTCTG
481  TTTGGTGGCTATGTTGCGTG GGAGATGGGCAAAGCTGAGG ACGGTCACGACTCTATGGCG GTTCAGATCGCGGACTCTTC
561  TGACTGGCCGCAGATGAAAG CGTGTGTTCTGGTTGTGAGC GACATCAAGAAGGACGTTTC TTCTACCCAGGGTATGCAGC
641  TGACCGTGGCGACCAGCGAA CTGTTTAAGGAGCGTATCGA ACACGTTGTTCCGAAGCGTT TTGAGGTTATGCGTAAGGCT
721  ATTGTTGAAAAGGACTTCGC AACCTTCGCTAAAGAAACCA TGATGGACTCCAACTCCTTT CACGCGACCTGCCTGGATTC
801  TTTCCCGCCGATTTTCTACA TGAACGACACCTCTAAACGT ATCATTTCCTGGTGCCACAC CATCAACCAGTTCTATGGTG
881  AAACTATCGTTGCGTACACC TTCGACGCGGGTCCGAACGC TGTTCTGTACTACCTGGCGG AAAACGAATCTAAGCTGTTT
961  GCATTCATTTACAAACTGTT CGGTTCTGTTCCGGGCTGGG ACAAAAAGTTCACCACTGAA CAGCTGGAGGCGTTTAACCA
1041 CCAGTTTGAGTCTTCTAACT TCACCGCGCGTGAGCTGGAC CTGGAACTGCAGAAAGACGT TGCGCGTGTTATCCTGACCC
1121 AGGTTGGTTCTGGCCCGCAG GAAACCAACGAGTCCCTGAT CGATGCGAAAACCGGTCTGC CGAAGGAATAA
```

Figure S4: Full ^{13}C NMR spectrum of (*R*)-mevalonate. Six single peaks corresponding to the six carbons of (*R*)-mevalonate are shown. Spectra were acquired on a 500 MHz Bruker AV(III)500 spectrometer equipped with a cryoprobe. Data were processed and analysed using ACD/NMR Processor version 12.01

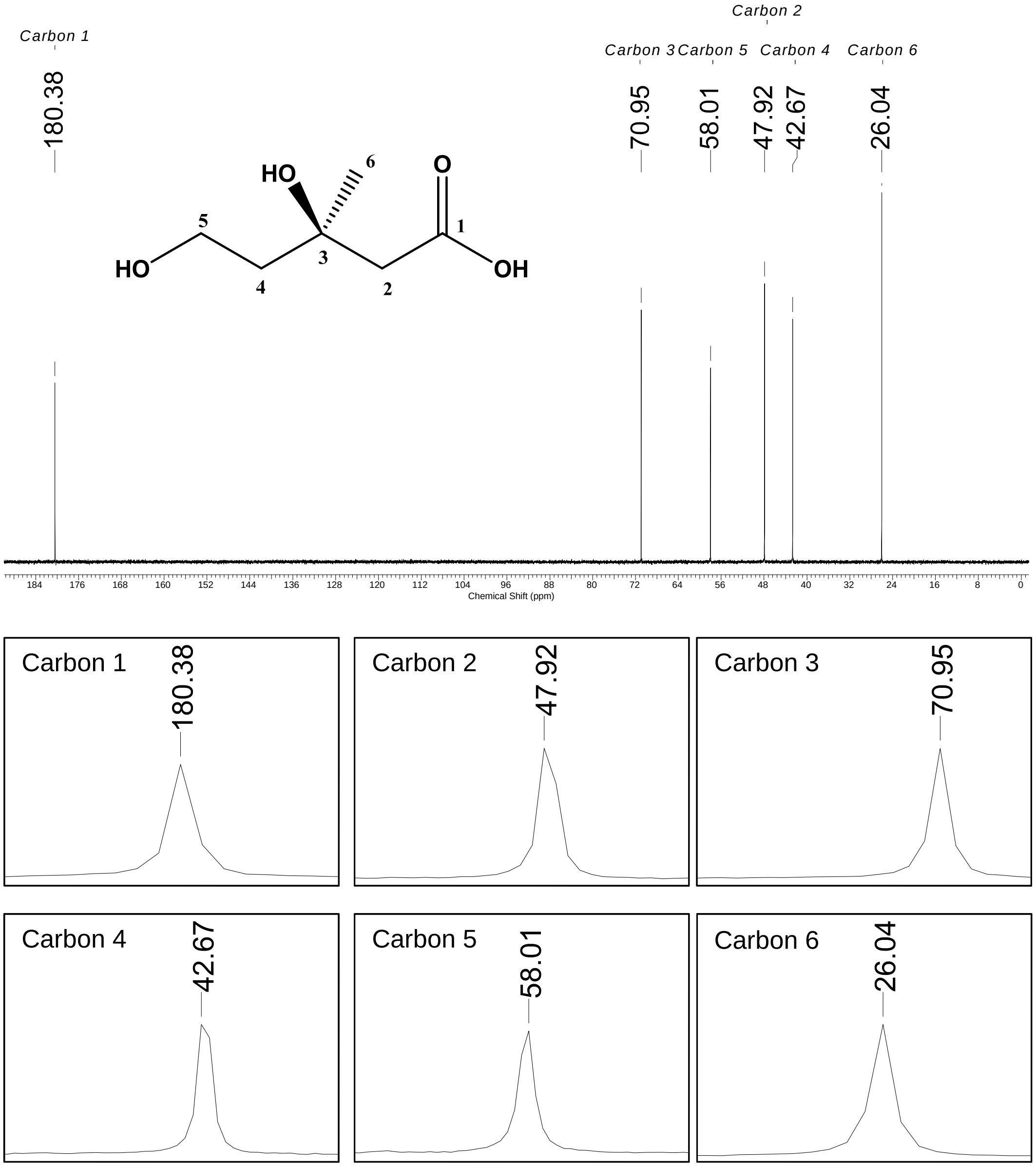

Figure S5: Full ^{13}C NMR spectrum of (*R*,*S*)-mevalonate-5-phosphate. Doublet peaks are observed corresponding to carbons 4 and 5 of (*R*,*S*)-mevalonate-5-phosphate. Spectra were acquired on a 500 MHz Bruker AV(III)500 spectrometer equipped with a cryoprobe. Data were processed and analysed using ACD/NMR Processor version 12.01

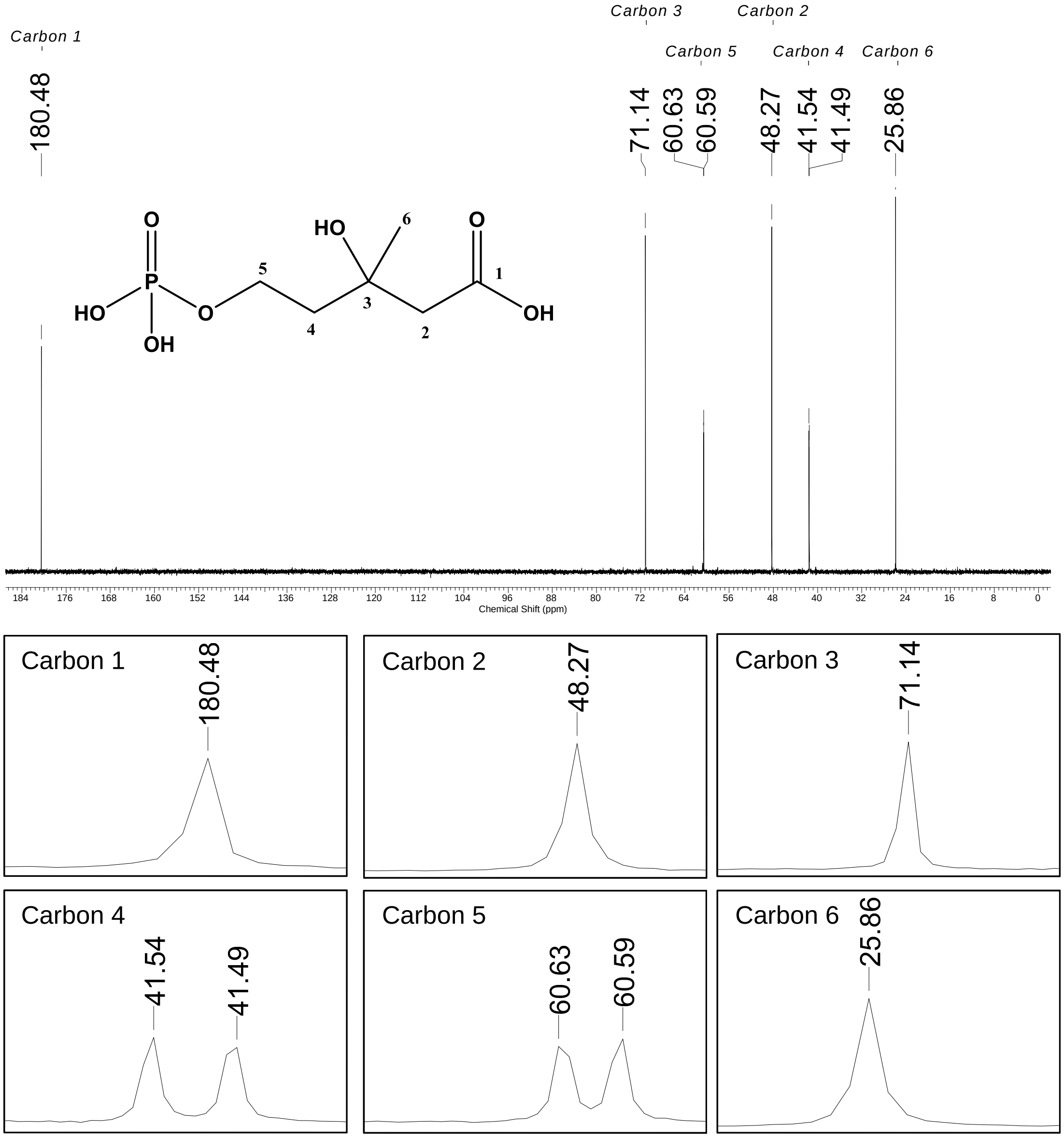

Figure S6: Full ^{13}C NMR spectrum of test reaction containing (*R*)-mevalonate and ^{put}*Pt*MVD (*Pt*M3K). Doublet peaks are observed corresponding to carbons 2 to 4 and 6. Additional peaks in the spectrum are from unreacted mevalonate, and phosphoenolpyruvate and pyruvate, which are present due to the system to regenerate ATP as previously described (5). Spectra were acquired on a 500 MHz Bruker AV(III)500 spectrometer equipped with a cryoprobe. Data were processed and analysed using ACD/NMR Processor version 12.01

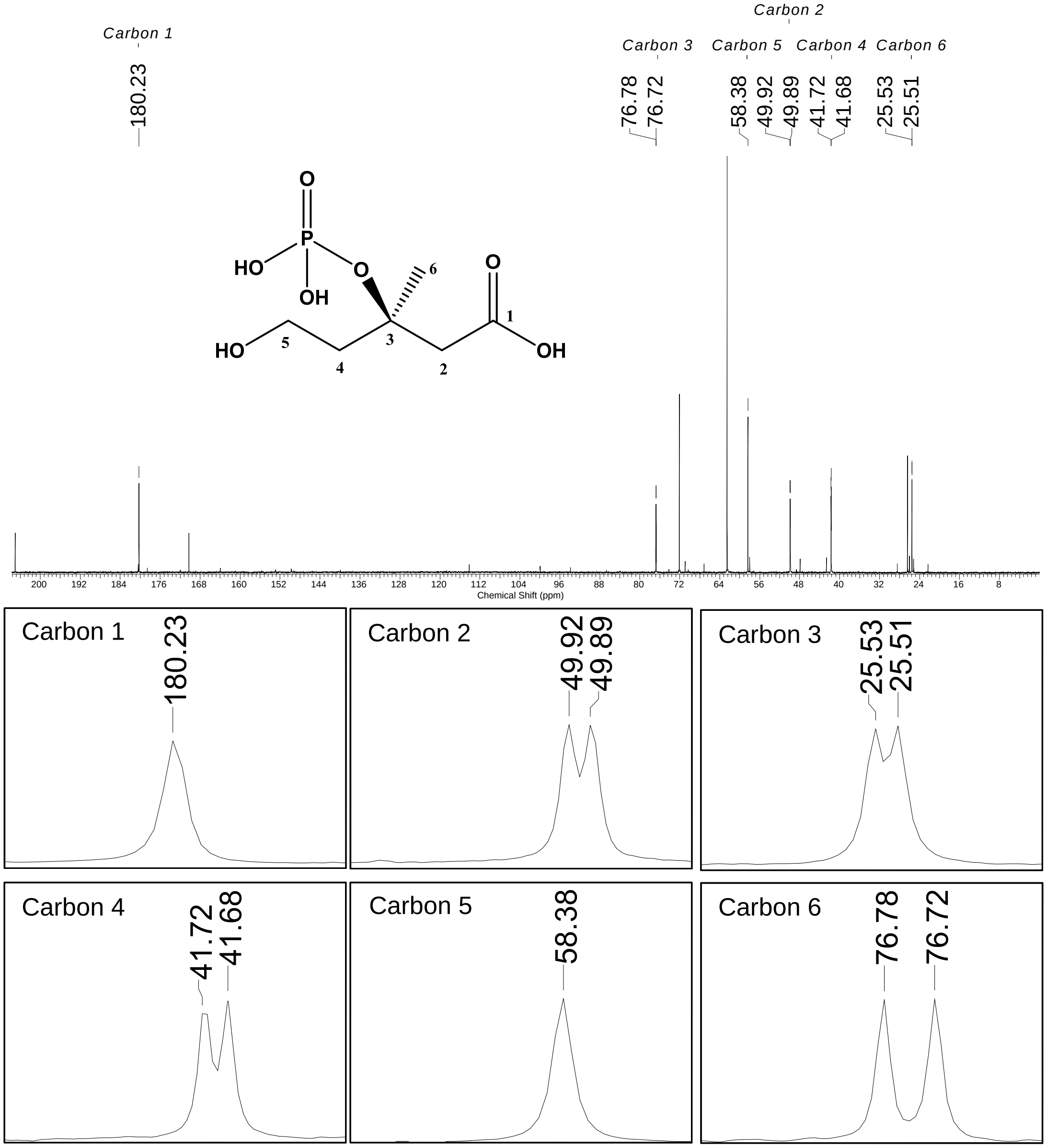

Table S7: Search for homologues of the newly discovered mevalonate-3-phopshate-5-kinase from *T. acidophilum* (5).

Sequences were searched with BLASTp (1) using the amino acid sequence of newly discovered *T. acidophilum* Ta0762 (GenBank (2) entry CAC11895) within nonredundant sequences database (July 2014). A 40% identity cut off was set. The *P. torridus* homologue is characterized by a level of identity of 55.5% (in bold).

	Organism	NCBI Reference	Identity (%)	Similarity (%)	E value
1	*Thermoplasma acidophilum*	WP_010901177	100	100	-
2	*Thermoplasma volcanium*	BAB60008	54.92	74.61	5.00E-72
3	*Thermoplasmatales archaeon A-plasma*	WP_021794774	52.88	68.06	2.00E-64
**4**	***Picrophilus torridus***	**WP_011177280**	**55.49**	**72.56**	**2.00E-56**
5	*Ferroplasma sp. Type II*	WP_021787503	46.56	69.31	4.00E-54
6	*Thermoplasmatales archaeon E-plasma*	WP_021792357	45.79	66.32	9.00E-53
7	*Ferroplasma acidarmanus*	WP_009886959	45.5	68.78	1.00E-51

References

1. **Altschul SF, Madden TL, Schaffer AA, Zhang J, Zhang Z, Miller W, Lipman DJ.** 1997. Gapped BLAST and PSI-BLAST: a new generation of protein database search programs. Nucleic Acids Res. **25:**3389-3402.
2. **Benson DA, Cavanaugh M, Clark K, Karsch-Mizrachi I, Lipman DJ, Ostell J, Sayers EW.** 2013. GenBank. Nucleic Acids Res. **41:**D36-42.
3. **Edgar RC.** 2004. MUSCLE: multiple sequence alignment with high accuracy and high throughput. Nucleic Acids Res. **32:**1792-1797.
4. **Marliere P.** January 2010. Production of alkenes by enzymatic decarboxylation of 3-hydroxyalkanoic acids. Patent WO2010001078A2.
5. **Vinokur JM, Korman TP, Cao Z, Bowie JU.** 2014. Evidence of a novel mevalonate pathway in archaea. Biochemistry **53:**4161-4168.
